# A Novel HMM-Based Method for Detecting Enriched Transcription Factor Binding Sites Reveals RUNX3 as a Potential Target in Pancreatic Cancer Biology

**DOI:** 10.1371/journal.pone.0014423

**Published:** 2010-12-22

**Authors:** Liron Levkovitz, Nir Yosef, Marvin C. Gershengorn, Eytan Ruppin, Roded Sharan, Yoram Oron

**Affiliations:** 1 Department of Physiology and Pharmacology, Sackler Faculty of Medicine, Tel Aviv University, Tel Aviv, Israel; 2 School of Computer Science, Tel Aviv University, Tel Aviv, Israel; 3 Clinical Endocrinology Branch, National Institute of Diabetes and Digestive and Kidney Diseases, National Institutes of Health, Bethesda, Maryland, United States of America; University of Nottingham, United Kingdom

## Abstract

**Background:**

Pancreatic adenocarcinoma (PAC) is one of the most intractable malignancies. In order to search for potential new therapeutic targets, we relied on computational methods aimed at identifying transcription factor binding sites (TFBSs) over-represented in the promoter regions of genes differentially expressed in PAC. Though many computational methods have been implemented to accomplish this, none has gained overall acceptance or produced proven novel targets in PAC. To this end we have developed DEMON, a novel method for motif detection.

**Methodology:**

DEMON relies on a hidden Markov model to score the appearance of sequence motifs, taking into account all potential sites in a promoter of potentially varying binding affinities. We demonstrate DEMON's accuracy on simulated and real data sets. Applying DEMON to PAC-related data sets identifies the RUNX family as highly enriched in PAC-related genes. Using a novel experimental paradigm to distinguish between normal and PAC cells, we find that RUNX3 mRNA (but not RUNX1 or RUNX2 mRNAs) exhibits time-dependent increases in normal but not in PAC cells. These increases are accompanied by changes in mRNA levels of putative RUNX gene targets.

**Conclusions:**

The integrated application of DEMON and a novel differentiation system led to the identification of a single family member, RUNX3, which together with four of its putative targets showed a robust response to a differentiation stimulus in healthy cells, whereas this regulatory mechanism was absent in PAC cells, emphasizing RUNX3 as a promising target for further studies.

## Introduction

Pancreatic adenocarcinoma (PAC) is one of the most aggressive cancers. Although 10th in incidence, it is the fourth leading cause of cancer deaths in the Western world. PAC is characterized by late diagnosis, rapid progression and extensive metastasis and is almost completely refractory to all therapeutic regimens. Although 10–15% of PAC tumors can be treated by partial pancreatectomy, the mean time between diagnosis and death is 3–6 months and the 5 year survival rate is under 5%. In the US, approximately 30,000 new cases are diagnosed each year and virtually the same number of PAC patients die each year of the disease[1], [2]. This grim picture makes this cancer a worthy subject for searching for novel therapeutic targets. However, published gene expression studies, so far, have failed to identify useful therapeutic targets.

Identification of transcription factors (TFs) involved in key biological processes and various pathological conditions, particularly cancer and inherited disorders, has gained popularity in recent years. TFs are master controllers of changes in expression of multiple genes and thus may serve as preferred targets for therapies of human diseases. A relatively large number of methods for identifying enriched TF binding sites (TFBSs) exist [3]–[5] but no single method has gained universal preference over the others.

Application of the state-of-the-art PRIMA algorithm [4] to data sets reflecting differential expression of genes in PAC pointed to ZNF350 as an important TF in PAC biology (unpublished). However, qRT-PCR experiments showed only modest changes in ZNF350 expression upon serum removal of PAC cells (see Fig. S1). In view of the importance of this methodology, we sought to develop a novel method aimed at achieving better predictive value in biological experiments.

A relatively large number of PAC gene expression studies have been performed, using both healthy and diseased pancreatic tissues and PAC lines in vitro. Brandt *et al.* [6] reviewed data from 10 expression studies and identified close to 1000 genes the expression of which change in PAC; 148 of these genes were identified in two or more studies. The list compiled by Brandt *et al*. includes genes that are expressed in a high proportion of PAC studies and had been associated with many types of cancers, such as Ras, Ink4, P53, etc. None, however, appear to explain the “catastrophic” [7] progression of this disease. Although individual proteins may serve as promising targets for drug development, the search for therapeutic targets in PAC has failed, so far, to produce novel promising drug leads. Conceptually, therapies targeted at TFs that are master regulators of expression of a large number of genes, are potentially more likely to affect cancer cell biology and are particularly attractive.

Here we have applied a new method, DEMON, for detecting enriched TFBSs and a new paradigm for comparing normal pancreatic and PAC cells. Applying DEMON to a PAC experimental data set predicted that binding sites for the RUNX subfamily of TFs are highly enriched in the pertinent differentially expressed gene sets. qRT-PCR confirmed RUNX3 as a differentially expressed TF. In conclusion, DEMON proved to be a helpful predictive tool in TFBSs analysis and, together with experimental results, suggests that RUNX3 may prove to be an important target TF in pancreatic cancer research.

## Results

### Detecting Enriched MOtifs in co-regulated geNes (DEMON)

Given a target set of promoters of co-regulated genes and a set of known TFBS motifs (represented as position weight matrices from the TRANSFAC database [8], see Methods), DEMON seeks motifs that appear in those promoters more frequently than expected by chance (i.e., motifs that are enriched in the target set). The algorithm utilizes a hidden Markov model (HMM) to describe the probabilistic process that generates the promoter sequences, and to estimate how likely it is that any given motif is enriched in the target set.

**Figure 1:**
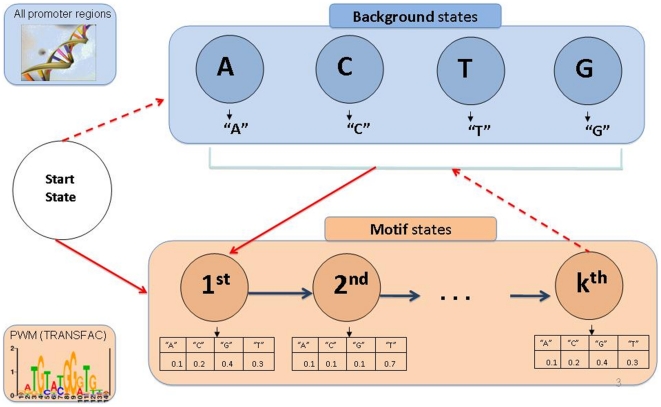
The structure of DEMON's HMM. The HMM is comprised of motif states (in pink), background states (in blue) and a start state. A background state is defined for each nucleotide (four states), and a motif state is defined for every position along the PWM corresponding to the TFBS of interest. The emission probabilities of the motif states are defined according to the PWM, and those of the background states are set to 1 for the corresponding nucleotide. Transition probabilities between the background states reflect the distribution of dinucleotides across all putative promoter regions in human. The transition probability from each motif state to the next is set to 1. Remaining transitions include moving to the background states (dashed arrows) or moving to the first motif state (solid arrows). These transitions are learned using the Baum-Welch algorithm.

**Figure 2:**
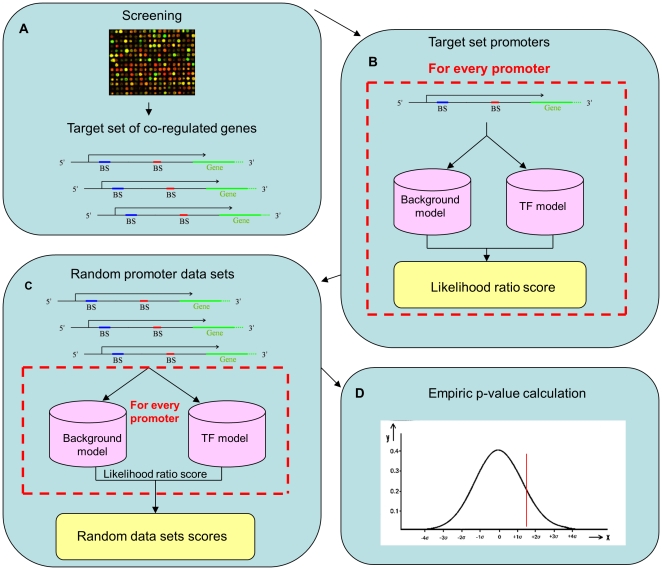
Schematic of the DEMON's algorithm work flow. a. Retrieving a list of co-expressed genes from high-throughput experiments. b. For each HMM-promoter pair a score is computed as the ratio between the probability to emit the promoter sequence using the TFBS HMM and the probability to emit the promoter sequence using a background HMM. The sum of scores for each TF is used for computing a single score reflecting the TF's overall abundance in the input promoter set. c. Randomly selecting 100 promoter data sets with the same size as the original data set. Scores are calculated as before for those data sets. d. Each TF is assigned with an empirical p-value defined as the percentage of random cases in which it scored higher.

Each HMM contains states for a unique motif, and background states that model inter-motif segments (Fig. 1). DEMON scores each promoter for the appearance of any given motif. This score reflects the probability that the sequence was generated based on the HMM describing the motif, vs. the probability that it was generated based on a simple background model. Given a target set of co-regulated genes, the scores of the promoters are summed up for each HMM, and compared to sums of scores obtained with random target sets. This comparison is used to assign a *p*-value for each motif that reflects its abundance in the promoter regions of the target set (see Fig. 2 and Methods).

### Performance evaluation on simulated and real data

To test our approach, we first benchmarked DEMON on simulated data. To this end we simulated sets of 100 random promoters, whose sequences were selected according to the background probability of dinucleotides in real promoter regions (Methods). We then planted a real motif in x% (10≤x≤90) of the promoters in each set (three instances of the motifs were planted in each promoter). We repeated this procedure for all the vertebrate position weight matrices (PWMs) in the TRANSFAC database [8] (see Methods).

**Figure 3:**
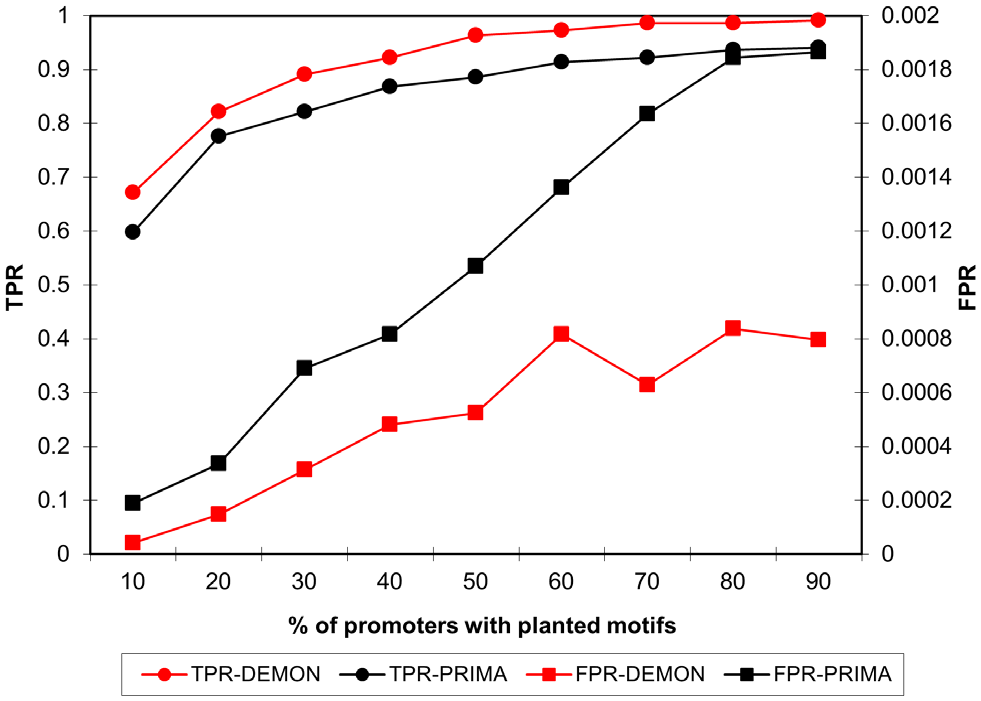
Results from the simulation benchmark. A comparison between DEMON's and PRIMA's performance on data sets with various percentage of promoters with planted motifs.

Figure 3 compares the performance of DEMON to that of the PRIMA algorithm. We chose PRIMA as a representative of a group of methods that use a hard threshold to identify putative appearances of motifs in any given promoter. Such methods may fail to identify “weak” occurrences of the motif and often do not take into account the actual number of occurrences of the motif (for instance, in PRIMA, promoters are categorized to those having 0, 1, 2, or more than 2 occurrences of the motif).

Evidently, in all cases DEMON achieves better results both in terms of specificity and sensitivity. We conducted additional simulations, varying the number of promoters in each set, or the number of planted motifs in each promoter. The results remained qualitatively similar (Figs S2 and S3).

Prima has a marginal advantage over DEMON on small data sets (for 30 promoters, DEMON false positive rate (FPR) is 0.0006 versus 0.0004 for PRIMA, see Fig. S3). However, these very low numbers make the FPR of both methods essentially equal.

Next, we compared the two methods on the recently published *Amadeus* metazoan benchmark, which is a collection of TF and microRNA target gene sets derived from high-throughput experiments (gene expression microarray and ChIP-on-chip experiments) [9]. We downloaded all human and mouse entries of this collection, where each entry contains a single TF and a list of target genes (ranging from 25 to 2238 genes).

Table 1 presents the results of DEMON and PRIMA over all the examined data entries. DEMON identified the true TF in 70.3% of the cases (where in 51.8% of the cases the true TF is ranked in first or second place) while PRIMA identified it in 55.5% of the cases (in 48.1% of the cases, the true TF is ranked in first or second place). Moreover, in 37% of the cases DEMON ranked the correct TF higher than PRIMA whereas PRIMA ranked the right TF higher than DEMON in only 18.5% of the cases.

**Table 1:** DEMON and PRIMA results on the Amadeus metazoan benchmark.

Organism	TF	# of genes	DEMON's rank	PRIMA's rank
Human	CREB	2334	**1**	2
	E2F4	201	**2**	3
	E2F4	79	2	**1**
	ER	495	**16**	–
	ETS1	1189	**5**	10
	E2F	264	1	1
	NF-Y	343	1	1
	HNF1α	206	1	1
	HNF4α	1471	**14**	–
	HNF6	211	**1**	–
	HSF1	328	–	**1**
	IRF/NF-κB	563	2	**1**
	Nanog	710	–	–
	NF- κB	270	–	**1**
	Nrf1	672	1	1
	OCT4	239	**1**	2
	p53	38	–	–
	SOX2	537	–	–
	SRF	172	1	1
	YY1	708	4	**1**
Mouse	FOXP3	1053	–	–
	IRF/NF-κB	322	1	1
	MEF2	25	**2**	–
	MyoD	102	**2**	–
	MyoD	102	–	–
	MyoG	106	–	–
	MyoG	78	(MyoD) –	–

The higher rank for each TF is marked in bold.

### Detecting TFs involved in the transcriptional regulation in PAC

We initially used a list of differentially expressed genes in PAC compiled by Brandt *et al.* [6] from 10 studies. We obtained from that list a smaller list of 45 genes that were identified as differentially expressed in 3 or more studies, of which 38 (30 that exhibited increased and 8 that exhibited decreased expression) matched our collection of human promoters (see Table S1). We analyzed this list using DEMON and found significant enrichment of 6 motifs, of which the most highly enriched motifs were for the RUNX sub-family of TFs (also called the AML sub-family). When we limited the consensus data set to the 30 genes that exhibited increased transcription, DEMON found significant enrichment of 8 motifs, of which the most highly enriched motifs were also for RUNX.

The TFs of the RUNX sub-family, are binding partners of heterodimeric transcriptional regulators denoted as CBFs (core-binding factors) of which the CBFa (RUNX) members bind directly to DNA and the two alternatively-spliced CBFb (also known as PEBP) members bind to the CBFa subunit and enhance its DNA binding [10]. It is noteworthy that PEBP appears as a third and a second most enriched TF, respectively (see Table 2).

**Table 2:** Top 10 TFBSs that were found by DEMON in the consensus data set and in the 30 consensus genes that exhibited increased transcription.

Consensus dataset (38 genes)		Consensus dataset (30 genes)	
TFBS	P-value	TFBS	P-value
**AML**	**0.00004**	**AML**	**0.000005**
**CP2**	**0.00019**	**PEBP**	**0.00008**
**PEBP**	**0.00025**	**PAX6**	**0.00028**
**PAX6**	**0.00035**	**CACCCBF**	**0.00038**
**MAZR**	**0.0008**	**CP2/LBP1C/LSF**	**0.00059**
**CACCCBF**	**0.0011**	**MAZR**	**0.0014**
NFY	0.0026	**PAX**	**0.0014**
AHR	0.0028	**LFA1**	**0.0016**
LFA1	0.0059	AHR	0.0042
PAX	0.0061	NFY	0.0055

Enriched TFBSs that pass a 0.05 FDR threshold appear in bold.

We used PRIMA to analyze the same lists, and found a significant enrichment of one motif, ZBRK1, also called ZNF350 (see Table S2). However, qRT-PCR experiments showed only modest changes in ZNF350 expression in PANC-1s upon serum withdrawal (unpublished results, see Fig. S1).

The three highly homologous human RUNX TFs (RUNX1, 2, and 3) have been implicated in developmental processes and, notably, in cancer. RUNX1 (also known as AML1) has been extensively documented as an important factor in hematopoiesis and in the etiology of acute myelogenous leukemia (for review see [11]). RUNX2 has been shown to be involved in bone development (for review see [12]) and RUNX3 was documented as an important TF in development of T-lymphocytes [13]–[15] and has been associated with the pathogenesis of several malignancies [16], including PAC [17], [18]. Hence, the DEMON analysis predicts that RUNX TF family members are top candidates responsible for altered transcription of genes in the PAC consensus data set.

### RUNX experimental validation

Most of the experimental data in cancer compare gene expression of cancer tissues with that of healthy tissues of human donors. This comparison filters out the variability of gene expression due to sex and age of the patient, stage of the disease, involvement of unrelated pathological conditions, different (cancer-targeted and other) drug therapies, as well as ethnic genetics and lifestyles. Thus, only the genes common to PAC on the background of all the above sources of variability are represented. It is noteworthy that Brandt's et al. [6] list of close to one thousand differentially expressed genes shrinks to 148 and 45 when one adds a requirement that it must appear in at least two or three studies, respectively.

To avoid the inter-patient variability, we chose to study the differential gene expression patterns observed in two cell types in culture: hIPCs, pancreatic precursor cells that outgrow from cultured human islets of Langerhans of healthy cadaveric donors, and PANC-1 cells, an established line of human PAC. Importantly, both types of cells undergo mesenchymal-to-epithelial transition (MET) and partially differentiate to a neuroendocrine phenotype when allowed to aggregate in serum-free medium [19], [20]. While hIPCs cease to proliferate and some of them die, PANC-1 cells continue to proliferate under these conditions.

The primary assumption of our paradigm is that the response to a differentiation stimulus will reveal changes of gene expression that distinguish normal from PAC cells. To the best of our knowledge, there is no proof in the literature that comparing processes in normal and cancer cells of similar origin under conditions that induce partial differentiation will yield insight into cancer-related gene expression. Continuous proliferation of cells in serum-free medium could be attributed to mutations of key genes (e.g., K-Ras). However, not all cancer cell traits (e.g. migration, invasiveness, stimulation of angiogenesis, resistance to cytotoxic agents) can be directly related to their ability to proliferate in the absence of growth factors. It is possible that this paradigm will yield genes that were missed in the traditional healthy vs. diseased tissue methods. We have, therefore, cultured both hIPCs and PANC-1 cells in serum-free medium for 24 h and compared changes in gene expression in both cell types. This comparison yielded a manually-curated set of 30 genes, whose expression changed significantly in one cell type and either did not change or exhibited change in the opposite direction in the other (see Table S3). We analyzed this set with DEMON (see Table S4). Although PEBP (CBFb) was only marginally enriched (p∼0.1) in this list, it appeared among the ten top TFBSs exhibiting the lowest p-values both in the lists derived by DEMON from consensus data sets (ranked 2nd and 3rd) and from the hIPCs vs. PANC-1 cells experiment data set (ranked 6th). This finding supported the prediction that RUNX sub-family members may be involved in PAC. Analysis of the same data set with PRIMA did not find any enriched motifs (see Table S5).

**Figure 4:**
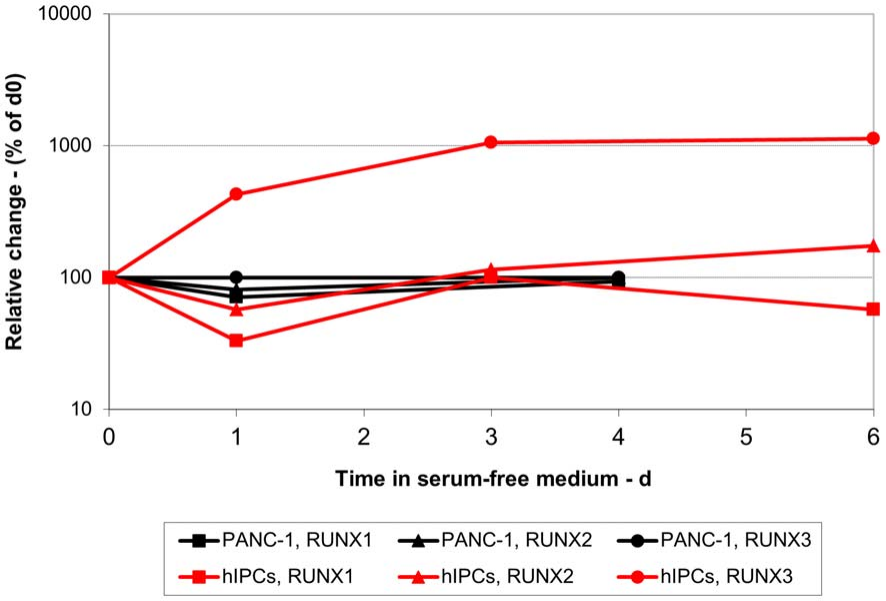
Kinetics of changes in expression of RUNX genes. hIPCs and PANC-1 cells were either cultured in serum-containing medium (t = 0) or for the indicated times in serum-free medium. RNA was extracted and qRT-PCR performed as described in Materials and Methods. Results are presented as % change in mRNA levels of the three RUNX genes as a function of time in serum-free medium.

To obtain experimental evidence for RUNX distinguishing between normal and PAC cells, we monitored expression of RUNX1, 2 and 3 mRNAs by qRT-PCR as a function of time of serum deprivation of hIPCs and PANC-1 cells (Fig. 4). There was little change in the expression of RUNX1 and 2 transcripts in either cell type. The expression of RUNX3, however, was markedly increased in a time-dependent manner in hIPCs while there was virtually no change in PANC-1 cells. It appears, therefore, that the expression of RUNX3 is regulated in hIPCs during differentiation but fails to respond to the differentiation stimulus in PANC-1 cells.

**Figure 5:**
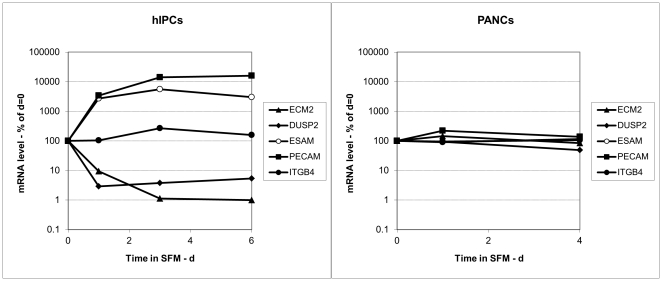
Kinetics of changes in expression of RUNX-controlled genes. A. hIPCs and B. PANC-1 cells were either cultured in serum-containing medium (t = 0) or for the indicated times in serum-free medium. RNA was extracted and qRT-PCR performed as described in Materials and Methods. Results are presented as % change in mRNA levels of the indicated genes as a function of time in serum-free medium.

To further validate this finding, we assayed in hIPCs the expression of five putative RUNX targets, ECM2, DUSP2, ESAM, PECAM, and ITGB4, that were chosen from a list of putative RUNX targets generated based on a procedure similar to the method described in [4]. Four of these mRNAs exhibited marked changes in expression (see Fig. 5A), while the fifth, ITGB4, exhibited only a transient two-fold increase. By comparison, the expression of these genes did not change in PANC-1 cells (see Fig. 5B). When the expression of the same genes was examined on the microarray data, none (including RUNX3) were high enough for meaningful analysis, confirming the superior sensitivity of qRT-PCR.

## Discussion

We have presented a new algorithm for detecting enriched TFBSs in a given set of promoters. The algorithm uses an HMM-based score to take into account all possible parses of a promoter sequence into binding sites and background nucleotides. It weighs in a principled manner all the potential binding sites along the promoter, making it possible to consider multiple weak binding sites that would not have passed a significance threshold. This is the first use of such a method for enrichment tests. We show that it outperforms a previous approach (PRIMA) to the problem, which uses a threshold to make binary decisions on actual binding sites.

Three aspects of the experimental results presented in this report appear to be of major importance. First, they experimentally validate the power of the DEMON analysis to predict TFs (and their target genes) from a small number of differentially expressed genes in PAC. Although DEMON proved to be superior to PRIMA in simulation experiments, its value can be proven only by its experimental predictive ability. In our case, the power of DEMON was not only validated for RUNX3, but also by the intrinsically consistent identification of CBFb, the heterodimeric partner(s) of the RUNX sub-family.

Second, our results strongly suggest that RUNX3 and its heterodimeric partner CBFb should be further investigated regarding their potential role(s) in PAC etiology. Aberrations in the expression of RUNX1 were identified in a significant proportion of leukemias [11]. RUNX2 and 3 genes have been extensively studied as developmental TFs. RUNX2 was shown to be crucial for bone and skeletal development [12]. RUNX3 was shown to be directly involved in the commitment of CD4+/CD8+ cells into CD8+ T-cells and in the maturation of dendritic T-cells [15], [21]. Some reports demonstrate the role of RUNX3 in the development of the sensory neuronal system [22], [23]. Hypermethylation of the RUNX3 promoter region has been correlated with various metastatic malignancies, such as breast, non-small cell lung, gastric, pancreatic, colorectal, or hepatocellular carcinomas [24]. Importantly, restoration of RUNX3 expression in cancer cell lines leads to apoptosis or decreased proliferation of cancer cells and to their differentiation [25]–[28]. These, and similar reports, established that RUNX3 appears to function as a tumor suppressor. They are further confirmed by our finding that untransformed mesenchymal hIPCs respond to a differentiation stimulus by increased RUNX3 transcription and proliferation arrest, while malignant PANC-1 cells appear to have lost this regulatory response and continue to proliferate. In human PAC, hypermethylation and loss of heterozygosity of RUNX3 were found in a large proportion of PAC tissues and correlated with worse prognosis [17], [18]. These findings place RUNX3 as another PAC-associated gene product. DEMON analysis, however, places RUNX and its partner, PEBP, as putatively very important TFs controlling the expression of many PAC-related genes.

Third, our results confirm the hypothesis that the differences between normal pancreatic and PAC cells are revealed following a differentiation stimulus. This assumption is further strengthened by a recent analysis of transcriptomes involved in cancer and development [29]. In proliferating hIPCs and PANC-1 cells, both exhibiting mesenchymal phenotypes [19], few RUNX3 transcripts are present (thresholds of 31.5 and 30 cycles, respectively). By 24 h in differentiation medium, however, the levels of RUNX3 mRNAs in hIPCs increased more than 1000-fold whereas there was virtually no response in PANC-1 cells. Likewise, putative RUNX3 target genes exhibited altered transcription in hIPCs but no changes in PANC-1 cells. Importantly, Li *et al*. [30] have found that RUNX3 is expressed only in islets and a proportion of PAC tissues. Our experimental data demonstrate that while RUNX3 mRNA expression may not be different in proliferating normal and PAC cells, its role is revealed only following differentiation stimulus, thus explaining the apparent disagreement between the findings of Wada *et al.* and Nomoto *et al.* [17], [18] and those of Li *et al*. [30].

Importantly, the differentiation-induced response of RUNX3 and its five putative targets in hIPCs cannot be gleaned from microarray analysis due to the absence of signal or their very low levels. Although PECAM1 and CBFA2T1 signals increased more than two-fold, their signals were too low to be significant. This justifies the use of computational methods, such as DEMON or PRIMA, to identify gene targets and their validation by the more sensitive qRT-PCR technique. Admittedly, qRT-PCR cannot reveal epigenetically-controlled regulations of cell phenotype.

Our results suggest loss of response of the RUNX3 gene in PAC and suggest further studies, such as investigation of methylation of its promoter, and a more extensive expression study of putative RUNX target genes.

## Materials and Methods

### The DEMON Algorithm

The DEMON algorithm uses HMMs for representing TFBSs. Each HMM is comprised of two types of states: motif states and background states (Fig. 1). A background state is defined for each nucleotide (four states), and a motif state is defined for every position along the PWM corresponding to the TFBS of interest. The emission probabilities of the motif states are defined according to the PWM, and those of the background states are set to 1 for the corresponding nucleotide. Transition probabilities between the background states reflect the distribution of dinucleotides across all putative promoter regions in human. The transition probability from each motif state to the next is set to 1. Remaining transitions include moving to the background states (Fig. 1, dashed arrows) or moving to the first motif state (Fig. 1, solid arrows). These transitions are learned using the Baum-Welch algorithm [31] (Supporting Information S1).

The inputs to DEMON are the list of genes of interest (Fig. 2a) and a set of TFBS motifs represented by PWMs. The output is a list of TFs whose binding sites are statistically over-represented in the promoter regions of the given list of genes.

As a first step, we build an HMM from every given PWM, and each HMM-promoter pair is assigned with a score reflecting the likelihood that the respective TFBS appears in the respective promoter region. This score is computed as the ratio between two values (Fig. 2b): (i) the probability to emit the promoter sequence using the TFBS HMM in Figure 1, and (ii) the probability to emit the promoter sequence using an HMM comprised solely of the background states. The probability values are computed using the Forward algorithm [32]. The pairwise scores are then being used for computing a single score for each TF, reflecting its overall abundance in the input promoter set. This score is defined as the sum over all scores assigned individually with each promoter.

In the second step, we use an empirical approach for evaluating the statistical significance of the overall likelihood scores computed for the TFs. We randomly select a similar number of promoters as in the original data set from the pool of all human promoter regions and compute a new score for each TF as before (Fig. 2c). We repeat this procedure 100 times, ending up with an empirical distribution of random likelihood scores. Each TF is then assigned with an empirical *p*-value defines as the probability to see the target set sum of scores, given the random sums which are assumed to be normally distributed (Fig. 2d). i.e., we compute the average and standard deviation of the random scores, and use the normal cumulative distribution function to compute the probability that an observation from a standard normal distribution will be higher than the target set sum of scores. The p-values are corrected for multiple hypotheses testing using the false discovery rate procedure [33]. We report all findings with false discovery rate under 5%.

### Data Acquisition and PRIMA implementation

We obtained a set of nucleotide distribution matrices that model vertebrate TFBSs from the TRANSFAC database (release 11.1) [8]. A total of 588 vertebrate matrices were downloaded from the database. The matrices were transformed to probability matrices that delineate the probability of each nucleotide to appear in each position in the TFBS. Since the database is redundant and some of the matrices describe similar TFBS, we clustered the matrices in a preprocessing step in a procedure similar to that used in [4]. To this end, we built a PWM *w* from each probability matrix *m*, and used a low pre-calculated threshold *t* to scan the human genome promoters. The threshold is computed using two sets of background promoters: (i) random promoters that are built based on the nucleotide distribution in all the promoters, (ii) randomly chosen segments of real promoters. The two sets are scanned by each PWM *w* and the threshold *t* is defined as the maximum between the 100^th^ highest score from each of the two background data sets (which implies an FPR of 0.01). Each subsequence that had a similarity score to the PWM *w* above the threshold *t* was marked as a putative instance of *w*. Then, each pair of matrices that *x*% of their appearances on the promoter set were overlapping was clustered and the matrix with the lower information content (i.e., the matrix which is less different from a uniform distribution) was removed. As the value of *x* grows, the clustering criterion becomes more stringent and the resulted matrices set grows, and vice versa. We used *x* = 0.2 to obtain a set of 219 matrices to use in our analysis.

We downloaded the complete set of human promoters from the UCSC Genome Browser database [34], [35]. Based on preliminary testing and recent studies claiming that most of the TFBSs in human promoters are located near the transcription start site [36], we define the promoter regions of the genes as the 500 bp sequence upstream to the transcription start site.

We have implemented PRIMA as described in [4].

### Cell cultures

Human islet-derived pancreatic precursor cells (hIPCs) were isolated and propagated in modified CMRL medium as previously described [20]. Human pancreatic adenocarcinoma cell line PANC-1 was purchased from American Tissue Type Collection and maintained in Dulbecco-modified minimal Eagle's Medium (DMEM) as previously described [20]. Partial differentiation of either cell type was achieved by culturing cells in serum-free medium, essentially as described previously [20]. Cells were grown and maintained in 95∶5% air:CO_2_ atmosphere at 37°.

### DNA Microarrays

Affymetrix GeneChip Human Genome U133 Plus 2.0 from microarray (catalog # 900466) was used, yielding 12,760 sequences. hIPCs were assayed in triplicate, each of a separate biological sample. PANC-1 cells were assayed in pentaplicate arrays, two from separate biological replicates and another biological replicate run in triplicate arrays. Each set was comprised of samples isolated from proliferating cells (t = 0, in 10% fetal bovine serum-containing medium) and cells after 24 h in serum-free (differentiation) medium. RNA samples were isolated and processed according to microarray manufacturer's instructions. The analysis, including normalization, was carried out using Affymetrix proprietary software.

Genes, whose expression changed at least two-fold upon 24 h in serum-free medium in either cell type, were compared. The selected set included genes that either exhibited changes in a single cell type, or genes that exhibited changes in the opposite direction. This set was subsequently used for DEMON analysis (Table S4).

### Quantitative real-time polymerase chain reaction (qRT-PCR) assay

Cells of either type were grown or maintained either in serum containing media (t = 0) or in serum-free media for the desired time period in 100 mm diameter tissue culture Petri dishes. RNeasy (Qiagen, Valencia, CA) RNA extraction kit was used throughout. RNA was quantified and its purity assessed by measurements of OD at 260/280 nm. Reverse-transcription was performed using the Applied Biosystems (Carlsbad, CA) kit, according to manufacturer's instructions. PCR was performed using TaqMan Assay-on-Demand kit, using the manufacturer's sequence-specific primers for each gene and Stratagene Mx3000P QPCR System. The five putative RUNX3-control genes were selected at random from the intersection of all putative RUNX3-control genes (∼1,000) and the list of primers reported to be involved in differentiation and available in the laboratory.

## Supporting Information

Supporting Information S1 A novel HMM-based method for detecting enriched transcription factor binding sites reveals RUNX3 as a potential target in pancreatic cancer biology. Calculating the HMM motif entry probability.
(0.14 MB DOC)

Figure S1 Changes in ZNF350 mRNA level in PANC-1 cells in serum-free medium.
(0.01 MB TIF)

Figure S2 Results from the simulation benchmark. A comparison between DEMON's and PRIMA's performance on data sets with various number of planted motifs.
(0.32 MB TIF)

Figure S3 Results from the simulation benchmark. A comparison between DEMON's and PRIMA's performance on data sets with various sizes.
(0.38 MB TIF)

Table S1 The list of 45 genes that were identified as differentially expressed in 3 or more studies out of a list of 10 studies compiled by Brandt, et al. [2].
(0.08 MB DOC)

Table S2 Top 10 TFBSs that were found by PRIMA in the consensus data set and in the 30 consensus genes that exhibited increased transcription.
(0.03 MB DOC)

Table S3 The list of 30 genes whose expression changed significantly in one cell type, hIPCs or PANC-1.
(0.05 MB DOC)

Table S4 Top 10 TFBSs that were found by DEMON in the PANC-1 vs. hIPCs data set.
(0.03 MB DOC)

Table S5 Top 10 TFBSs that were found by PRIMA in the PANC-1 vs. hIPCs data set.
(0.03 MB DOC)
