# Supplementary material for: A Novel HMM-Based Method for Detecting Enriched Transcription Factor Binding Sites Reveals RUNX3 as a Potential Target in Pancreatic Cancer Biology
Source: PLoS One. 2010 Dec 22;5(12):e14423. doi: 10.1371/journal.pone.0014423 (PMC3008686; doi:10.1371/journal.pone.0014423)
Supplement: Supporting Information S1 — A novel HMM-based method for detecting enriched transcription factor binding sites reveals RUNX3 as a potential target in pancreatic cancer biology. Calculating the HMM motif entry probability. (0.14 MB DOC) [file pone.0014423.s001.doc]

## A novel HMM-based method for detecting enriched transcription factor binding sites reveals RUNX3 as a potential target in pancreatic cancer biology

**Supporting information**

**Calculating the HMM motif entry probability**

The probability to move from any state of the HMM to the state that represents the first position of the motif (i.e., the motif entry probability) is an unknown parameter. It cannot be calculated directly from the data since the TFBS locations in the promoters are unknown. We use the Baum-Welch algorithm [1] that maximizes the log-likelihood of the promoter sequence, to study this probability. The training set for the algorithm is the set of all the genome promoters.

Typically, the algorithm considers the constraint that the sum of all transition probabilities going out from a state should be equal to 1. In our case, there is an additional constraint, which requires that the motif entry probability *p* will remain the same in all relevant states. This causes dependencies between the different transition probabilities. i.e., the probability to move from each one of the background states to the first motif state is equal to the probability to move from the last motif state to the first motif state, and to the probability to move from the start state to the first motif state. Those dependencies obligate some changes to the algorithm equations.

As explained in [1], maximizing

$$\text{Q(}\theta\text{|}\theta^{\text{t}}\text{)=}\sum_{k} \sum_{b} E_{k}(b)\log e_{k}(b)+\sum_{k} \sum_{l} A_{kl}\log(a_{kl})$$

increases the sequence likelihood. In our case, we do not want to learn the emission probabilities, so our only parameters are the transition probabilities, $a{}_{kl}$. Moreover, the transition probabilities are either dependent on *p* or constant, and since *p* is the only parameter we would like to learn, it is sufficient to differentiate $\text{Q(}\theta\text{|}\theta^{\text{t}}\text{)}$as a derivative of *p.* The resulting *p* that maximizes the likelihood of the sequence is

$$p=\frac{\sum_{k|A_{kl}=p} A_{kl}}{\sum_{k} \sum_{l'} A_{kl'}}$$

Using this update criterion, the sequence likelihood increases iteratively until a local maximum is reached.

|  |  |  |  |
| --- | --- | --- | --- |
|  |  |  |  |
|  |  |  |  |
|  |  |  |  |
|  |  |  |  |
|  |  |  |  |
|  |  |  |  |
|  |  |  |  |
|  |  |  |  |
|  |  |  |  |
|  |  |  |  |
|  |  |  |  |
|  |  |  |  |
|  |  |  |  |
|  |  |  |  |
|  |  |  |  |
|  |  |  |  |
|  |  |  |  |
|  |  |  |  |
|  |  |  |  |
|  |  |  |  |
|  |  |  |  |
|  |  |  |  |
|  |  |  |  |
|  |  |  |  |
|  |  |  |  |
|  |  |  |  |
|  |  |  |  |
|  |  |  |  |
|  |  |  |  |
|  |  |  |  |
|  |  |  |  |
|  |  |  |  |
|  |  |  |  |
|  |  |  |  |
|  |  |  |  |
|  |  |  |  |
|  |  |  |  |
|  |  |  |  |
|  |  |  |  |
|  |  |  |  |
|  |  |  |  |
|  |  |  |  |
|  |  |  |  |
|  |  |  |  |
|  |  |  |  |

|  | |  | |
| --- | --- | --- | --- |
|  |  |  |  |
|  |  |  |  |
|  |  |  |  |
|  |  |  |  |
|  |  |  |  |
|  |  |  |  |
|  |  |  |  |
|  |  |  |  |
|  |  |  |  |
|  |  |  |  |
|  |  |  |  |

|  |  |
| --- | --- |
|  |  |
|  |  |
|  |  |
|  |  |
|  |  |
|  |  |
|  |  |
|  |  |
|  |  |
|  |  |
|  |  |
|  |  |
|  |  |
|  |  |
|  |  |
|  |  |
|  |  |
|  |  |
|  |  |
|  |  |
|  |  |
|  |  |
|  |  |
|  |  |
|  |  |
|  |  |
|  |  |
|  |  |
|  |  |
|  |  |

|  | | |
| --- | --- | --- |
|  |  |  |
|  |  |  |
|  |  |  |
|  |  |  |
|  |  |  |
|  |  |  |
|  |  |  |
|  |  |  |
|  |  |  |
|  |  |  |
|  |  |  |

|  | | |
| --- | --- | --- |
|  |  |  |
|  |  |  |
|  |  |  |
|  |  |  |
|  |  |  |
|  |  |  |
|  |  |  |
|  |  |  |
|  |  |  |
|  |  |  |
|  |  |  |

References

1. Baum LE, Petrie T, Soules G, Weiss N (1970) A maximization technique occurring in the statistical analysis of probabilistic functions of markov chains. The Annals of Mathematical Statistics 41: 164-171.

2. Brandt R, Grutzmann R, Bauer A, Jesnowski R, Ringel J, et al. (2004) DNA microarray analysis of pancreatic malignancies. Pancreatology 4: 587-597.
