# Supplementary figures and images for: A Novel HMM-Based Method for Detecting Enriched Transcription Factor Binding Sites Reveals RUNX3 as a Potential Target in Pancreatic Cancer Biology
Source: PLoS One. 2010 Dec 22;5(12):e14423. doi: 10.1371/journal.pone.0014423 (PMC3008686; doi:10.1371/journal.pone.0014423)

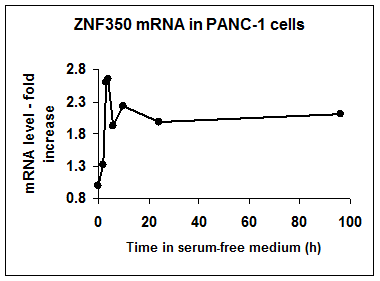

Supplement: Figure S1 — Changes in ZNF350 mRNA level in PANC-1 cells in serum-free medium. (0.01 MB TIF) [file pone.0014423.s002.tif]

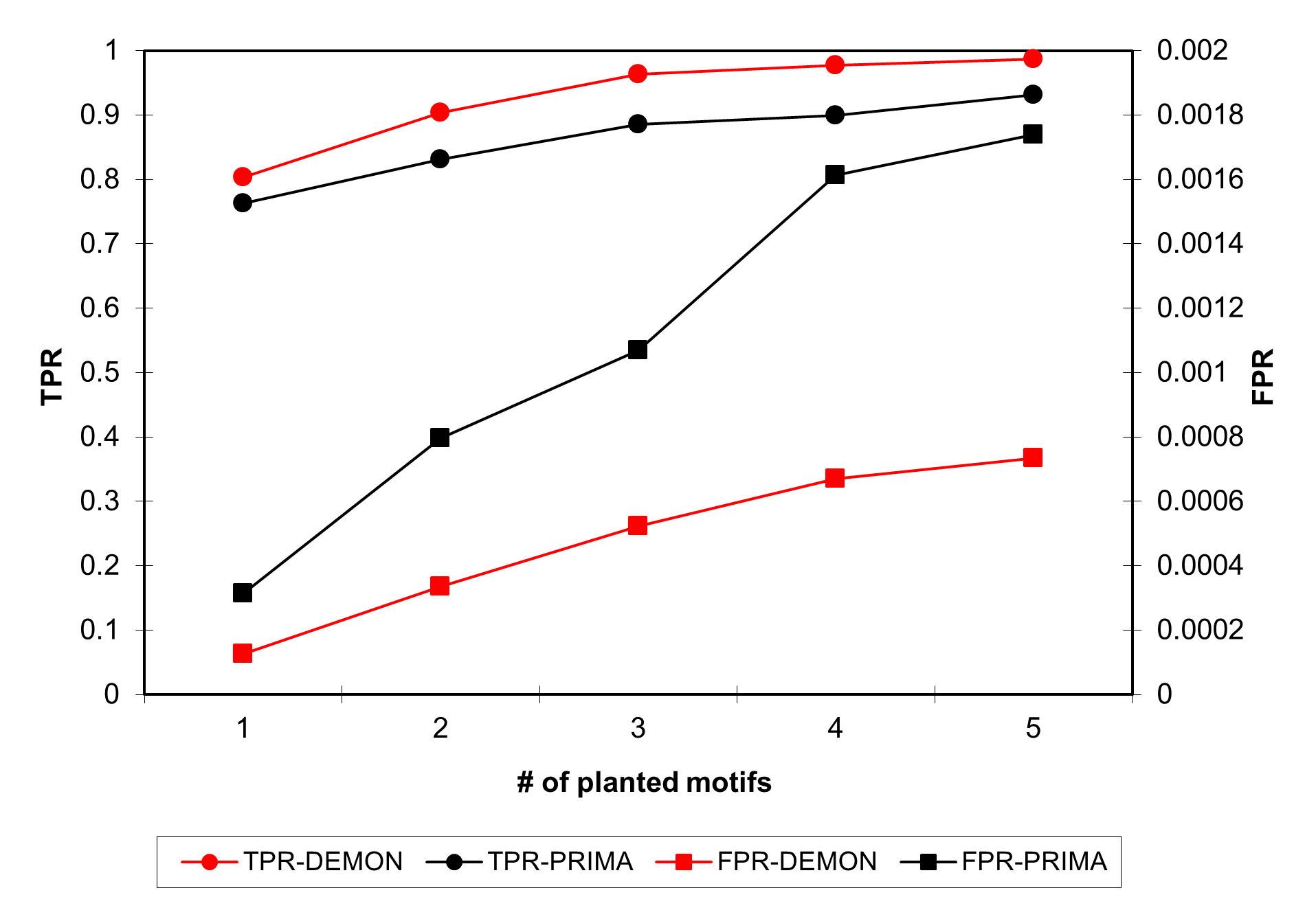

Supplement: Figure S2 — Results from the simulation benchmark. A comparison between DEMON's and PRIMA's performance on data sets with various number of planted motifs. (0.32 MB TIF) [file pone.0014423.s003.tif]

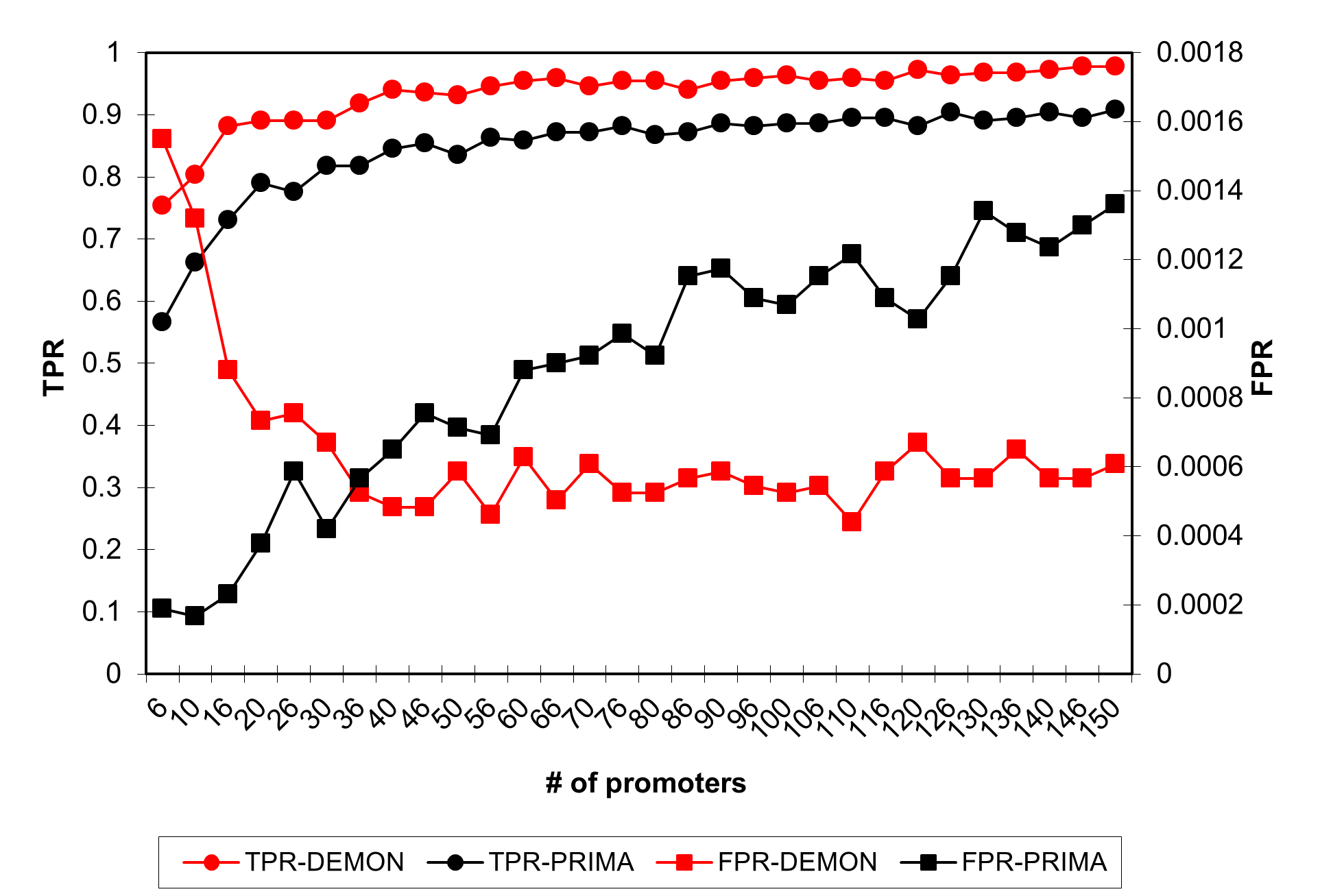

Supplement: Figure S3 — Results from the simulation benchmark. A comparison between DEMON's and PRIMA's performance on data sets with various sizes. (0.38 MB TIF) [file pone.0014423.s004.tif]
