# Supplementary material for: A Novel HMM-Based Method for Detecting Enriched Transcription Factor Binding Sites Reveals RUNX3 as a Potential Target in Pancreatic Cancer Biology
Source: PLoS One. 2010 Dec 22;5(12):e14423. doi: 10.1371/journal.pone.0014423 (PMC3008686; doi:10.1371/journal.pone.0014423)
Supplement: Table S1 — The list of 45 genes that were identified as differentially expressed in 3 or more studies out of a list of 10 studies compiled by Brandt, et al. [2]. (0.08 MB DOC) [file pone.0014423.s005.doc]

| **Gene ID** | **Gene Name** | **Overexpressed in tumor tissue in more than 2 studies** | **Overexpressed in normal tissue in more than 2 studies** |
| --- | --- | --- | --- |
| **NM_005980** | **S100P** | **√** |  |
| **NM_005564** | **LCN2** | **√** |  |
| **NM_000700** | **ANXA1** | **√** |  |
| NM_005562 | LAMC2 | **√** |  |
| **NM_003311** | **PHLDA2** | **√** |  |
| NM_002659 | PLAUR | **√** |  |
| **NM_003979** | **RAI3** | **√** |  |
| **NM_003064** | **SLPI** | **√** |  |
| **NM_000574** | **DAF** | **√** |  |
| **NM_005620** | **S100A11** | **√** |  |
| **NM_012101** | **TRIM29** | **√** |  |
| **NM_001747** | **CAPG** | **√** |  |
| XM_039877 | MUC5AC | **√** |  |
| **NM_005737** | **ARL7** | **√** |  |
| **NM_001910** | **CTSE** | **√** |  |
| BC005256 | CAV2 | **√** |  |
| **NM_001197** | **BIK** | **√** |  |
| **NM_001067** | **TOP2A** | **√** |  |
| **NM_006516** | **SLC2A1** | **√** |  |
| **NM_006142** | **SFN** | **√** |  |
| **NM_004363** | **CEACAM5** | **√** |  |
| NM_003816 | ADAM9 | **√** |  |
| **NM_000422** | **KRT17** | **√** |  |
| **NM_001175** | **ARHGDIB** | **√** |  |
| **NM_005423** | **TFF2** | **√** |  |
| NM_021910 | FXYD3 | **√** |  |
| **NM_002276** | **KRT19** | **√** |  |
| **NM_004004** | **GJB2** | **√** |  |
| AK027130 | IMUP | **√** |  |
| **NM_013451** | **FER1L3** | **√** |  |
| **NM_002245** | **KCNK1** | **√** |  |
| **NM_002026** | **FN1** | **√** |  |
| **NM_001311** | **CRIP1** | **√** |  |
| **NM_000598** | **IGFBP3** | **√** |  |
| **NM_000584** | **IL8** | **√** |  |
| **NM_001827** | **CKS2** | **√** |  |
| **NM_000213** | **ITGB4** | **√** |  |
| **NM_002257** | **KLK1** |  | **√** |
| **NM_014573** | **MAC30** |  | **√** |
| **NM_182848** | **CLDN10** |  | **√** |
| **NM_013230** | **CD24** |  | **√** |
| **NM_006280** | **SSR4** |  | **√** |
| **NM_138938** | **PAP** |  | **√** |
| **NM_001482** | **GATM** |  | **√** |
| **NM_000928** | **PLA2G1B** |  | **√** |

**Table S1** The list of 45 genes that were identified as differentially expressed in 3 or more studies out of a list of 10 studies compiled by Brandt *et al* [2]*.* The 38 genes that matched our collection of human promoters are marked in bold.
