# Supplementary material for: A Novel HMM-Based Method for Detecting Enriched Transcription Factor Binding Sites Reveals RUNX3 as a Potential Target in Pancreatic Cancer Biology
Source: PLoS One. 2010 Dec 22;5(12):e14423. doi: 10.1371/journal.pone.0014423 (PMC3008686; doi:10.1371/journal.pone.0014423)
Supplement: Table S2 — Top 10 TFBSs that were found by PRIMA in the consensus data set and in the 30 consensus genes that exhibited increased transcription. (0.03 MB DOC) [file pone.0014423.s006.doc]

| Consensus data  set (38 genes) | | Consensus data  set (30 genes) | |
| --- | --- | --- | --- |
| TFBS | P-value | TFBS | P-value |
| **ZBRK1** | **9.24E-05** | SREBP-1 | 0.0004 |
| SREBP-1 | 0.001482 | ZBRK1 | 0.000456 |
| PEBP | 0.002505 | PEBP | 0.000839 |
| Nrf-2 | 0.005442 | CACD | 0.003818 |
| CACD | 0.01251 | SREBP 0.005618 | 0.005618 |
| SREBP | 0.013007 | AP-1 | 0.007457 |
| ATF6 | 0.016623 | Nrf-2 | 0.008682 |
| AP-1 | 0.017048 | Ncx | 0.011281 |
| Ncx | 0.021391 | Pax | 0.012317 |
| MyoD | 0.021927 | LF-A1 | 0.019873 |

**Table S2** Top 10 TFBSs that were found by PRIMA in the consensus data set and in the 30 consensus genes that exhibited increased transcription. Enriched TFBSs that pass a 0.05 FDR threshold appear in bold.
