# Supplementary material for: A Novel HMM-Based Method for Detecting Enriched Transcription Factor Binding Sites Reveals RUNX3 as a Potential Target in Pancreatic Cancer Biology
Source: PLoS One. 2010 Dec 22;5(12):e14423. doi: 10.1371/journal.pone.0014423 (PMC3008686; doi:10.1371/journal.pone.0014423)
Supplement: Table S3 — The list of 30 genes whose expression changed significantly in one cell type, hIPCs or PANC-1. (0.05 MB DOC) [file pone.0014423.s007.doc]

| **Gene Id** | **Gene Name** |
| --- | --- |
| NM 001769 | CD9 |
| NM 001827 | CKS2 |
| NM 005438 | FRA1 |
| NM 000584 | IL8 |
| NM 000212 | ITGB3 |
| NM 000213 | ITGB4 |
| NM 002224 | ITPR3 |
| NM 002658 | PLAU |
| NM 001005376 | PLAUR |
| NM 005620 | S100A11 |
| NM 002997 | SDC1 |
| NM 000593 | TAP1 |
| NM 004240 | TRIP10 |
| NM 006000 | TUBA1A |
| NM 015201 | BOP1 |
| NM 001947 | DUSP7 |
| NM 001005915 | ERBB3 |
| NM 005239 | ETS2 |
| NM 001987 | TEL |
| NM 013962 | NRG1 |
| NM 032945 | TNFR6A |
| NM 001025242 | IRAK1 |
| NM 006562 | LBX1 |
| NM 004635 | MAPKAPK3 |
| NM 015670 | SENP3 |
| NM 003733 | OASL |
| NM 183234 | RAB27A |
| NM 002960 | S100A3 |
| NM 006142 | SFN |
| NM 002204 | ITGA3 |

**Table S3** The list of 30 genes whose expression changed significantly in one cell type, hIPCs or PANC-1.
