# Supplementary material for: A Novel HMM-Based Method for Detecting Enriched Transcription Factor Binding Sites Reveals RUNX3 as a Potential Target in Pancreatic Cancer Biology
Source: PLoS One. 2010 Dec 22;5(12):e14423. doi: 10.1371/journal.pone.0014423 (PMC3008686; doi:10.1371/journal.pone.0014423)
Supplement: Table S4 — Top 10 TFBSs that were found by DEMON in the PANC-1 vs. hIPCs data set. (0.03 MB DOC) [file pone.0014423.s008.doc]

| **PANC-1 vs. hIPCs data set (30 genes)** | | |
| --- | --- | --- |
| **TFBS (Transfac ID)** | **Name** | **P-value** |
| **M00986** | **Churchill** | **0.000174** |
| M00706 | TFII-I | 5.91E-04 |
| M01118 | WT1 | 8.48E-04 |
| M00189 | AP-2 | 2.20E-03 |
| M00800 | AP-2 | 7.93E-02 |
| M00984 | PEBP | 9.83E-02 |
| M00485 | Nkx2-2 | 1.12E-01 |
| M01100 | LRF | 1.42E-01 |
| M00320 | Muscle TATA box | 1.50E-01 |
| M00665 | Sp3 | 3.07E-01 |

**Table S4** Top 10 TFBSs that were found by DEMON in the PANC-1 vs. hIPCs data set. The significantly enriched TFBS is marked in bold.
