# Supplementary material for: A Novel HMM-Based Method for Detecting Enriched Transcription Factor Binding Sites Reveals RUNX3 as a Potential Target in Pancreatic Cancer Biology
Source: PLoS One. 2010 Dec 22;5(12):e14423. doi: 10.1371/journal.pone.0014423 (PMC3008686; doi:10.1371/journal.pone.0014423)
Supplement: Table S5 — Top 10 TFBSs that were found by PRIMA in the PANC-1 vs. hIPCs data set. (0.03 MB DOC) [file pone.0014423.s009.doc]

| **PANC-1 vs. hIPCs data set (30 genes)** | | |
| --- | --- | --- |
| **TFBS (Transfac ID)** | **Name** | **P-value** |
| M01105 | ZBRK1 | 0.000456 |
| M00973 | E2A | 0.00503 |
| M00706 | TFII-I | 0.0102 |
| M00273 | R | 0.0141 |
| M00490 | Bach2 | 0.02053 |
| M00444 | VDR | 0.024329 |
| M01122 | ZNF219 | 0.024665 |
| M00196 | Sp1 | 0.02636 |
| M01118 | WT1 | 0.030582 |
| M00744 | POU1F1 | 0.034608 |

**Table S5** Top 10 TFBSs that were found by PRIMA in the PANC-1 vs. hIPCs data set. There were no significantly enriched TFBS found in this data set.
